# RETRACTION: Influence of Continuous Nursing on Surgical Site Wound Infection and Postoperative Complication for Colorectal Cancer Patients With Stoma: A Meta‐Analysis

**DOI:** 10.1111/iwj.70276

**Published:** 2025-03-03

## Abstract

**RETRACTION:** LiuX.‐J. , HanJ. , and SuX. , “Influence of Continuous Nursing on Surgical Site Wound Infection and Postoperative Complication for Colorectal Cancer Patients With Stoma: A Meta‐Analysis,” International Wound Journal 21, no. 4 (2024): e14480, 10.1111/iwj.14480.38083831 PMC10958097

The above article, published online on 11 December 2023, in Wiley Online Library (http://onlinelibrary.wiley.com/), has been retracted by agreement between the journal Editor in Chief, Professor Keith Harding; and John Wiley & Sons Ltd. Following an investigation by the publisher, all parties have concluded that this article was accepted solely on the basis of a compromised peer review process. The editors have therefore decided to retract the article. The authors did not respond to our notice regarding the retraction.

**RETRACTION:** X.‐J. Liu , J. Han , and X. Su , “Influence of Continuous Nursing on Surgical Site Wound Infection and Postoperative Complication for Colorectal Cancer Patients With Stoma: A Meta‐Analysis,” International Wound Journal 21, no. 4 (2024): e14480, 10.1111/iwj.14480.38083831 PMC10958097

The above article, published online on 11 December 2023, in Wiley Online Library (http://onlinelibrary.wiley.com/), has been retracted by agreement between the journal Editor in Chief, Professor Keith Harding; and John Wiley & Sons Ltd. Following an investigation by the publisher, all parties have concluded that this article was accepted solely on the basis of a compromised peer review process. The editors have therefore decided to retract the article. The authors did not respond to our notice regarding the retraction.

